# The Updated NICE Guidance Exposed the Serious Flaws in CBT and Graded Exercise Therapy Trials for ME/CFS

**DOI:** 10.3390/healthcare10050898

**Published:** 2022-05-12

**Authors:** Mark Vink, Alexandra Vink-Niese

**Affiliations:** 1 Independent Researcher, 1096 HZ Amsterdam, The Netherlands; 2 Independent Researcher, 30159 Hannover, Germany; vinkniese.alexandra@gmail.com

**Keywords:** CFS, chronic fatigue syndrome, eminence-based medicine, evidence-based medicine, ME, NICE, post-infectious disease

## Abstract

The British National Institute for Health and Care Excellence (NICE) recently published its updated guidelines for myalgic encephalomyelitis/chronic fatigue syndrome (ME/CFS). NICE concluded, after an extensive review of the literature, that graded exercise therapy (GET) is harmful and should not be used, and that cognitive behavioural therapy (CBT) is only an adjunctive and not a curative treatment. Leading proponents of the cognitive behavioural model (CBmodel) find it difficult to accept this paradigm shift. In, for example, an article in *The Lancet*, they try to argue that the new NICE guideline is based on ideology instead of science. In this article we reviewed the evidence they used to support their claims. Our analysis shows that the trials they used in support suffered from serious flaws which included badly designed control groups, relying on subjective primary outcomes in non-blinded studies, including patients in their trials who didn’t have the disease under investigation or had a self-limiting disease, selective reporting, outcome switching and making extensive endpoint changes, which created an overlap in entry and recovery criteria, using a post-hoc definition of recovery which included the severely ill, not publishing results that contradict their own conclusion, ignoring their own (objective) null effect, etc. The flaws in these trials all created a bias in favour of the interventions. Despite all these flaws, treatments that are said to lead to recovery in reality do not lead to objective improvement. Therefore, these studies do not support the claim that CBT and GET are effective treatments. Moreover, the arguments that are used to claim that NICE was wrong, in reality, highlight the absence of evidence for the safety and efficacy of CBT and GET and strengthen the decision by NICE to drop CBT and GET as curative treatments for ME/CFS.

## 1. Introduction

The National Institute for Health and Care Excellence (NICE) published its updated guidelines on the diagnosis and management of myalgic encephalomyelitis/chronic fatigue syndrome (ME/CFS) in October 2021 [1,2]. NICE concluded that ME/CFS is a complex multisystem chronic medical condition and that it is important that people remain in their energy envelope when undertaking activity of any kind. Additionally, any program based on fixed incremental increases in physical activity or exercise, for example graded exercise therapy (GET), should not be offered for the treatment of ME/CFS. NICE emphasized that cognitive behavioural therapy (CBT) is not a treatment or cure but that it might be offered as a supportive therapy. These conclusions are in line with the conclusion from the prestigious American Institute of Medicine (IOM, now the National Academy of Medicine) in 2015 and the Dutch Health Council in 2018 [3,4]. Leading proponents of the cognitive behavioural model (CBmodel) from Norway, Denmark and The Netherlands [5] recently wondered in a commissioned article in *The Lancet* entitled *New NICE guideline on chronic fatigue syndrome: more ideology than science*, how it can be that NICE has changed its course, as according to them, there is now more evidence for the efficacy of CBT and GET than in 2007 when the previous guideline was published [6]. In this article we will review the evidence presented by Flottorp et al. in *The Lancet* [5] in regards to the safety and efficacy of CBT and GET, the main characteristic of ME/CFS, and the use of subjective outcomes in non-blinded studies, to see if there is any merit in it and if NICE should have come to a different conclusion. Other important evidence presented by Flottorp et al. is reviewed in Appendix A of this article.

## 2. Background

Many doctors still think that ME/CFS is a psychosomatic illness. This view has been reinforced by Surawy’s et al. cognitive behavioural model (CBmodel) from 1995 and a similar model by Vercoulen et al. from 1998. According to these models, patients’ illness beliefs, coping strategies and other behavioural factors are key factors in both the onset and perpetuation of the condition [7,8]. Patients avoid exercise to reduce their symptoms, but as a result they become deconditioned. This results in chronic disability and the false belief that one has an ongoing incurable illness. Cognitive behavioural therapy (CBT) tackles the illness-related cognitions and behaviours that work to maintain and perpetuate symptoms. Graded exercise therapy (GET) is used to improve the levels of physical activity and conditioning of the patient. On top of that, the graded exposure tackles the view that exercise and overexertion are feared stimuli. Ultimately, the aim of CBT and GET for ME/CFS is recovery from symptoms through psychological change and reconditioning [9]. This has led to the predominant recommendation of CBT and GET as effective treatments. However, reviews by Song and Jason in 2005 [10] and Sunnquist and Jason in 2018 [11] showed that if patients are selected who fulfil more stringent case definitions, so that ME/CFS patients are selected instead of those with a fatiguing psychiatric illness, then they do not possess the behavioural characteristics targeted by the CBmodel. Also, Geraghty et al. [12] concluded that “the CBM [CBmodel] is not fit for purpose, as it poorly reflects the accounts given by patients and it ignores the wealth of evidence showing biological, immune and neurological dysfunction in ME/CFS”. As noted by the developers of the model themselves [7], “the observations [upon which the model is based] have been made during treatment [of 100 patients] and require confirmation by objective measurement techniques”. Although this was written in 1995, this objective evidence has so far not been presented. 

### 2.1. A Major Shift in Interpretation. How Could This Happen?

Flottorp et al. [5] state in their article in *The Lancet* that “it is remarkable that recommendations in the 2021 NICE guideline differ substantially from the previous one, and do not include CBT and GET as means to treat CFS/ME. The new guideline presents strengthened evidence, but a major shift in interpretation. How could this happen?”

One of the reasons for this *major shift in interpretation* might be the following. Prins et al. [13] conducted the largest CBT study in the Netherlands so far and concluded in 2001 that CBT is an effective treatment for adult patients with ME/CFS. Stulemeijer et al. [14] came to a similar conclusion in 2005, but for adolescents. NICE and many other medical professionals in those days believed the authors of these studies when they concluded that CBT is an effective treatment. However, neither study mentions that they also used an objective outcome measure. Wiborg et al. published a study in 2010 [15] in which they analysed the actometer results from three different CBT studies, which included the aforementioned two studies. The third study (Knoop et al., 2008 [16]) is a study by Knoop (one of the authors of Flottorp et al.) who, together with the leading authors of the other two studies, was also part of Wiborg et al. The analysis of the actometer results by Wiborg et al. found that CBT did not lead to objective improvement in all three studies. NICE didn’t know in 2007 that these studies had used an objective outcome measure. Moreover, NICE also didn’t know back then that the objective outcome measure from Prins et al. and Stulemeijer et al. contradicted the conclusion of these two studies, because the results of these measures were published in 2010, three-years after NICE published its guidelines in 2007.

### 2.2. Studies Used by Flottorp et al. to Support Their Claim

Flottorp et al. [5] also state that the change in recommendation is remarkable because since the publication of the previous guideline in 2007, “more randomised trials and systematic reviews have provided additional evidence supporting these recommendations”. The authors use the following studies to support their claim [5].

- The Cochrane CBT review by Price et al., (2008) [17]
- The amended Cochrane exercise review by Larun et al., (2019) [18]
- A meta-analysis of CBT and GET studies by Castell et al., (2011) [19]
- The PACE trial by White et al., (2011) [20]
- The FITNET study by Nijhof et al., (2012) [21]
- Gotaas et al., (2021) [22]

### 2.3. Analysis of These Studies

#### 2.3.1. Cochrane CBT and GET Reviews and a Meta-Analysis of CBT and GET

Flottorp et al. [5] first of all criticize the NICE guideline committee for not including the Cochrane CBT and GET reviews. Secondly, they state that “the guideline committee decided not to include Cochrane reviews, because the review did not report one of the crucial outcomes determined by the committee, namely mortality. Mortality was not an outcome in any of the trials considered, however, because it is not relevant in studies of CBT and GET for CFS/ME”.

However, according to the NICE guideline committee itself in Appendix G of the updated NICE ME/CFS guideline, entitled *Evidence reviews for the non-pharmacological management of ME/CFS* [23], the Cochrane CBT review, for example, was excluded for the following reason. “One Cochrane review of cognitive behavioural therapy (Price 2008) included study populations where not all participants had ME/CFS. Additionally, the review did not include all critical outcomes specified in this review protocol, including cognitive function, pain, sleep quality, activity levels, exercise performance and mortality”. However, the committee also stated that “all included studies within these [three] reviews [Cochrane reviews of CBT, exercise interventions and Chinese medicinal herbs] were cross-checked for eligibility for inclusion in this review” [23]. In other words, the Cochrane reviews were not excluded for the reason mentioned by Flottorp et al.. Also, the committee didn’t use the conclusion from the Cochrane reviewers, but checked the included studies themselves, which is what a guideline committee should do.

Almost all of the studies in the meta-analysis by Castell et al., (2011) [19] are also included in the aforementioned two Cochrane reviews [17,18] and also in the independent reanalyses of those Cochrane reviews [24,25,26]. Notable exceptions are a GET study by Gordon et al., (2010) [27], which had only 11 participants in its treatment group, a study of family-focused CBT by Chalder et al., (2010) [28] and the aforementioned CBT study with a waiting list control group by Knoop et al., (2008) [16], which is one of several Dutch studies that concluded that CBT is effective but (initially) did not publish their objective outcome (actometer). When the authors themselves published their objective outcome many years later, it was reported that CBT did not lead to objective improvement [15].

The pilot study by Gordon et al. from 2010 [27] examined the efficacy in twenty-two adolescents aged 13–18 years of a four-week inpatient program of either graded aerobic exercise training or a progressive resistance-training programme, both for five days per week. Eighteen percent dropped out of the graded aerobic exercise training and 0% from the resistance training program. Gordon et al. concluded that “no intervention was significantly better than the other for any outcome”. They also noted that “interpretation of this data should be done with caution however, as it is limited by the small sample size and the lack of a non-exercising, standard care control group”.

The aforementioned study by Knoop et al., was evaluated by NICE (Appendix G of the NICE guidance, page 32) [23]. Just like the study of family focused CBT by Chalder et al., (Appendix G of the NICE guidance, page 13) [23]. This study by Chalder et al., (*n* = 63), in which only 69% of patients had CFS according to the Fukuda criteria, found no difference between family-focused CBT or psychoeducation for CFS in 11- to 18-year-olds [28]. These three studies got a methodological quality score of 39% (Gordon et al.), 57% (Chalder et al.) and 37% (Knoop et al.) from Castell et al. [19]. In comparison, the PACE trial by White et al., (2011) [20], was rated at 75%, although it seems that the reviewers were not aware of all the endpoint changes and other problems of that study, which we will discuss later.

One of the studies in the meta-analysis by Castell et al., but also in the Cochrane CBT review, is a study by Lloyd et al. [29]. This study is of particular interest for the current discussion because the authors found that neither immunological therapy (dialyzable leukocyte extract) nor CBT (alone or in combination) provided greater benefit than nonspecific treatment regimens. The graded exercise regime in the CBT group programme did not lead to significantly increased levels of non-sedentary physical activity either. Lloyd et al. concluded that “our results do not support the hypothesis that CFS would be adequately treated by dialyzable leukocyte extract and/or CBT”.

Flottorp et al. [5] use the 2019 amended Cochrane Review of exercise for ME/CFS by Larun et al. [18] to support their claim of efficacy, but they do not mention that this review was subsequently reanalyzed. The independent reanalysis [26] highlighted the continuing problems with the amended Cochrane Review. For example, Larun et al. did not report the objective data which, on analysis, showed the absence of objective improvement after GET. Flottorp et al. also do not mention that The Cochrane Library has announced that the exercise review is being overhauled as a consequence of these ongoing problems [30]. Why they didn’t mention this is unclear because one of the authors of Flottorp et al., (Brurberg) [5] was also one of the four authors of Larun et al.. Flottorp et al. also do not mention that The Cochrane Library has put an editorial note on the Cochrane CBT review by Price et al. from 2008, stating that “the review is no longer current. It should not be used for clinical decision-making” [31].

Flottorp et al. [5] do not mention the systematic review by Ahmed et al. from 2019 either [32]. The reviewers concluded that “the findings of this systematic review do not support the claim that CBT and GET are effective treatments for ME/CFS patients, due to methodological flaws and biases found in the studies that are investigated in this”. One of the included studies was the PACE trial by White et al., which Flottorp et al. use as evidence for their claim of efficacy of CBT and GET. According to Ahmed et al., PACE suffered from performance and detection bias, reporting bias (selective reporting) and other biases. The authors also concluded that “Prins et al., (2001) and White et al., (2011) [and two other studies] scored a high risk of bias in three or four categories, making them the most biased studies”.

#### 2.3.2. The PACE Trial

The PACE (“Pacing, graded Activity, and Cognitive behaviour therapy: a randomised Evaluation”) trial [20] is the biggest CBT and GET trial for ME/CFS (*n* = 641). Serious questions about research methodology in the PACE trial and other studies of CBT and graded exercise therapy for ME/CFS, were raised by an editorial by Geraghty and by the *Journal of Health Psychology*’s special issue on the PACE trial [33,34]. Professor Edwards [35] concluded in an article in that special issue that the researchers of the PACE trial failed “to address the key design flaw of an unblinded study with subjective outcome measures, apparently demonstrating a lack of understanding of basic trial design requirements. The failure of the academic community to recognise the weakness of trials of this type suggests that a major overhaul of quality control is needed”.

Two of the many problems with the non-blinded PACE trial are the reliance on subjective primary outcomes and the fact that the authors made an extensive number of endpoint changes which created an overlap in entry and recovery criteria. Consequently, one could be ill and recovered at trial entry, that is, before receiving any treatment. This is even more of a problem as 13.3% of patients were classed as ill enough to take part, but were also already classed as recovered according to one or two of the primary outcomes before they had received any treatment [36]. Yet patients who already fulfil recovery criteria at baseline should be excluded from a properly conducted trial. The endpoint changes also created a four-fold increase in recovery rates of CBT and GET [37]. Without those changes, there would have been no difference with the control groups (specialist medical care and adaptive pacing treatment or APT). The researchers also ignored the null effect at long-term follow-up (LTFU) as well as the fact that their treatments did not lead to objective improvements [36,37]. Moreover, in their cost-effectiveness analysis, they ignored that more patients were unable to work and were reliant on illness benefits after treatment with CBT and GET than before treatment with it [38,39]. The lack of effect on employment and benefit status was one of the points raised in a response by Dr. Shepherd, the medical advisor of the British ME Association [40]. The PACE trial authors responded to that in the following manner: “recovery from illness is a health status, not an economic one, and plenty of working people are unwell, while well people do not necessarily work. Some of our participants were either past the age of retirement or were not in paid employment when they fell ill. In addition, follow-up at 6 months after the end of therapy may be too short a period to affect either benefits or employment. We therefore disagree with Shepherd (2013) that such outcomes constitute a useful component of recovery in the PACE trial” [41]. Moreover, the main principal investigator of the PACE trial recently wrote the following about the lack of effect of CBT and GET on employment and benefit status in *The HealthSense* newsletter from spring 2022. “These treatments made no significant difference to the numbers working six months later, but there may have been alternative explanations for this, such as not having a job to return to” [42]. We don’t think that many people will dispute the fact that the economy and other factors may play a role as well. However, the PACE trial concluded that CBT and GET led to improvement in 60% and recovery in 22% of participants. Consequently, there should have been a substantial increase in the number of patients who were working and not a decrease. Moreover, a study by Stevelink et al. [43], which included professor Chalder, one of the PACE trial’s principal investigators, concluded in 2021 that the CBT and GET studies should pay more attention to work related outcomes, yet it ignored the PACE trial’s null effect on those outcomes.

#### 2.3.3. The FITNET Study

The Fatigue In Teenagers on the interNET (FITNET) trial [21] was a non-blinded study that compared an Internet-based CBT program for adolescents with that of usual care (UC). An analysis of this study found many problems with the design and the reporting of the outcomes [44]. For example, the usual care control group was badly designed, as it is unclear what treatment they received. For example, 57% received more than one treatment but it is unclear which treatments these were, 66% received CBT, some received unspecified inpatient treatment, 22% received physical treatment (mostly GET), 24% received an unspecified alternative treatment and 10% did not receive any treatment at all. Also, according to the authors, ME/CFS was diagnosed or confirmed by a paediatrician specialising in ME/CFS, yet only 28% of patients had an infection as the triggering factor of their illness [21], even though ME/CFS is also known as post-viral (or post-infectious) fatigue syndrome. Moreover, during the trial, three patients were given new diagnoses (school phobia, personality disorder or gender identity disorder, and post-traumatic stress disorder due to family violence), yet they were not excluded from the analysis even though patients who do not have the disease under investigation should be excluded from a properly conducted trial.

The FITNET trial also selected only mildly affected patients, as shown by the baseline physical functioning scores of 60.7 (14.5, FITNET) and 56.8 (20.9, UC). On top of that, many patients had only been ill for less than a year as the duration of symptoms at entry (months) shows: 16.0 (6–84, FITNET) 19.0 (6–108, UC) and many patients suffered from depression (21%, FITNET; 13%, UC) and anxiety (13%, FITNET; 9%, UC). This also means that quite a few people were included who had been ill for less than four years and many adolescents recover spontaneously within the first four years of being ill with ME/CFS, according to a review [44]. These figures (physical function, depression, and anxiety scores) also suggest that at baseline the treatment and control group were not evenly matched with many patients in the treatment group having better physical function, and more patients were suffering from mental health problems in the CBT group than in the control group. This is also the very subset of patients one would expect to respond positively to CBT, as a meta-analysis by Tolin et al. found CBT to be the most effective therapy for anxiety and depression [45]. Moreover, Reeves et al., co-authored by one of the FITNET investigators, concluded in 2003 that patients with a comorbid medical or psychiatric condition that may explain the chronic fatigue state should be excluded from CFS research studies because overlapping pathophysiology may confound findings specific to CFS [46]. Why the FITNET trial did not do this is unclear.

On top of that, FITNET used a post-hoc definition of recovery that included the severely ill and they did not publish their actometer results, which might suggest that these did not back up their claims of efficacy. Despite all of these biases, the trial still found no significant difference in recovery rates at LTFU, the trial’s primary goal [44]. Moreover, in a response to Kindlon, the authors state that “actual physical activity as measured by actigraphy is not likely to be the mediator of reduction in fatigue” [47]. The authors thereby acknowledge in an indirect way the absence of objective improvement after their treatment. In view of all these flaws and the absence of objective improvement, it is impossible to know if any improvement was a result of the intervention or not.

#### 2.3.4. The Non-Blinded Study

This non-blinded study by Gotaas et al. [22] used subjective outcomes and compared 16 weeks of standard CBT with graded activity/exercise to eight weeks of interpersonal CBT (I-CBT). The authors concluded that the positive effect of CBT on physical function and fatigue and of I-CBT on physical function in CFS outpatients with mild to moderate disease was sustained for one year. However, the difference in physical functioning scores between I-CBT and the control group was only 6.8 points. This is lower than the 10 points the authors had chosen themselves as the minimal important difference for their treatment to be effective. Therefore, the authors should have concluded that I-CBT is not effective.

Moreover, patients in the control group received no treatment (waiting list), and a study without a properly designed control group doesn’t correct for regression to the mean [48,49,50]. This problem became even more of an issue because the control group was not scored at (1-year) follow-up. Consequently, at that point the trial became a non-controlled study, and it became impossible to tell if any improvement was down to their treatment or not. According to the protocol [51], professor Chalder, one of the principal investigators of the PACE trial, was their advisor. PACE concluded something similar at follow-up when it stated that “the main finding of this long-term follow-up study of the PACE trial participants is that the beneficial effects of the rehabilitative CBT and GET therapies on fatigue and physical functioning observed at the final 1 year outcome of the trial were maintained at long-term follow-up” [52]. However, their main findings at long-term follow-up were that there was no difference in efficacy between CBT, GET, APT and no treatment, and that none of them were effective [53].

The mean duration of illness in Gotaas et al. was 4.5 years (I-CBT, range: 0.5–14) and 5.3 years (CBT, range: 0.8–13). This might indicate that the two groups were not evenly matched, as people in the control group had been ill for longer. It also means that quite a few people were included who had been ill for less than three years, and the chances of spontaneous recovery in adults are the highest within the first two to three years, according to an extensive review [54].

According to the authors themselves, there was a substantive difference in dropout rate between the standard CBT group compared to the I-CBT group (20.5% vs. 6%). Also, after “effective” treatment, fatigue scores showed that patients were still ill enough to enter the PACE trial according to the post-intervention (20.7, I-CBT; 18.7 CBT) and one year follow-up scores (19.3, I-CBT; 19.7, CBT).

One of the references of Gotaas et al. is a study by Núñez et al. [55] which compared multidisciplinary treatment combining CBT, GET and pharmacological treatment with usual treatment. It found that at twelve months follow-up, the interventions did not improve health-related quality of life scores and led to worse physical function and bodily pain scores. Núñez et al. concluded that “the combination of CBT and GET is ineffective and not evidence-based and may in fact be harmful”.

Also, Gotaas et al. did not publish their quality-of-life scores, one of their three subjective primary outcomes, and they also did not publish their objective physical functioning scores (VO_2_max), the study’s only objective outcome. Other CBT and GET studies that did not publish their objective outcomes initially showed that these interventions did not lead to objective improvement [15,24,25,26,36,37,47]. In view of all these flaws, it is impossible to know if any improvement resulted from the interventions or not.

### 2.4. Subjective Outcomes in Non-Blinded Studies

According to Flottorp et al. [5], “it is uncontroversial that a diagnosis of CFS/ME rests upon subjective symptoms. But paradoxically [the NICE guideline committee] decided that evidence from clinical trials of CBT and GET showing improvement in subjective symptoms would be considered unreliable. Given the first premise, subjective symptoms are the most valid endpoints, and interventions improving these symptoms are treatments, not only “symptom management”. Flottorp et al. also state that [5], “NICE ignored…patient-reported outcomes, such as fatigue, pain, and quality of life”.

There are a number of issues with these statements. First of all, patients have been saying for a long time that CBT and GET are not effective. The only way then to find out if CBT and GET are effective or not, when researchers disagree with patients, is by using objective outcomes.

Secondly, why do researchers ignore what patients say, after stating that it is all about what patients say (“subjective symptoms are the most valid endpoints”)?

Thirdly, an influential systematic review by Whiting et al. from 2001 [56], concluded the following about the use of subjective outcomes in CBT and GET trials. “Outcomes such as ‘improvement’, in which participants were asked to rate themselves as better or worse than they were before the intervention began, were frequently reported. However, the person may feel better able to cope with daily activities because they have reduced their expectations of what they should achieve, rather than because they have made any recovery as a result of the intervention. A more objective measure of the effect of any intervention would be whether participants have increased their working hours, returned to work or school, or increased their physical activities”.

Fourthly, colleagues from professor Knoop from the Dutch knowledge centre of fatigue concluded “that one has to be very careful with using self-report questionnaires as measures for actual activity level: [as] none of the self-report questionnaires had strong correlations with the Actometer” [57].

Fifthly, the unreliability of subjective outcomes in non-blinded trials has been documented extensively [58,59,60,61,62,63].

Sixthly, another problem with outcomes assessed via questionnaires in a non-blinded trial is "response-shift bias". This occurs when an intervention leads individuals to change their evaluation standard with regard to the dimension measured, leading the therapist (and often also the patient) to conclude erroneously that the treatment has worked [44,63]. This is even more of a problem when the therapy used, in this case CBT, aims to modify participants’ beliefs and perception of their symptoms.

Seventhly, there is a low correlation between objective and subjective activity measurements [64] not only in chronically ill but also in healthy people [65].

Eighthly, Lilienfeld et al. [63] concluded that non-blinded trials should not rely on subjective primary outcomes but use either objective primary outcomes alone or in combination with subjective ones as a methodological safeguard against erroneous inference of efficacy in its absence.

Additionally, as concluded by the BRANDO project (Bias in Randomised and Observational studies), which amongst others included Stanford professor Ioannidis, “as far as possible, clinical and policy decisions should not be based on trials in which blinding is not feasible and outcome measures are subjectively assessed” because lack of blinding is “associated with an average 13% exaggeration of intervention effects…Therefore, trials in which blinding is not feasible should focus as far as possible on objectively measured outcomes” [62].

As found by the reanalysis of the amended Cochrane exercise review [26], analysis of objective outcomes from three CBT and GET trials for ME/CFS confirmed the unreliability of subjective outcomes in non-blinded studies, as shown by the following examples:

1. In Jason et al., (2007) [66], there was a substantial difference in subjective physical functioning scores at baseline between the exercise and control group, yet objectively there wasn’t (six-minute walk test or 6MWT);
2. In Moss-Morris et al., (2005) [67], after GET, physical functioning subjectively improved by 30%, yet objectively deteriorated by 15% (CPET);
3. In the aforementioned PACE trial by White et al., (2011) [20], the released individual participant data showed that 20% of participants whose physical functioning improved subjectively had deteriorated objectively (6MWT) [38,68,69].

Finally, a systematic review by Haywood et al., (2011) [70] of patient-reported outcome measures (PROMs) in ME/CFS, found poor quality of the reviewed PROMs, which included the Chalder Fatigue Scale, with clear discrepancies between what is measured in research and which outcomes are relevant to patients. They also concluded that “the poor quality of reviewed PROMs combined with the failure to measure genuinely important patient outcomes suggests that high quality and relevant information about treatment effect is lacking”. Moreover, “rarely do developers work collaboratively with patients to develop and evaluate PROMs to ensure their relevance, acceptability and appropriateness”. Even though many trials have been published since, that is still lacking, and researchers continue to claim efficacy in its absence by using PROMs, which are irrelevant to patients.

Subjective outcomes which are more relevant to patients are, for example, the quality-of-life scores and the chronic fatigue syndrome symptom count. Three trials from the Cochrane CBT review [17] as well as the PACE trial recorded quality-of-life scores as found by the reanalysis of that review [25]. CBT was not more effective in Prins et al., (2001) [13] for the quality-of-life than no treatment (natural course). In Jason et al., (2007) [66], the quality-of-life scores after CBT improved by 5% less than after relaxation. Group CBT did not bring about improvement in quality-of-life in O’Dowd et al., (2006) [71]. Moreover, the net improvement of the quality-of-life scores (EQ-5D) in the PACE trial by White et al., (2011) [20] at the end of treatment, which is the time Flottorp et al. [5] prefer to look at results, was minimal (2.3% after GET over APT and 0.5% after CBT over APT) [39]. This is despite the fact that according to the baseline quality-of-life scores, groups were not evenly matched and patients in the GET (0.52) and CBT (0.54) groups were less disabled than in the APT group (0.48). Also, the quality-of-life scores after treatment with GET (0.60) and CBT (0.61) [39] were similar to the score (0.60) for people with five or more chronic health conditions and were still worse than in cerebral thrombosis (0.62), rheumatoid arthritis and angina (0.65), acute myocardial infarction (AMI) (0.66) [72], MS (0.67), lung cancer (0.69), stroke (0.71) or ischaemic heart disease (0.72) (higher scores indicating a better quality-of-life) [73].

The chronic fatigue syndrome symptom count is another relevant patient reported outcome as the CBmodel claims that CBT and GET lead to recovery. The PACE trial by White et al. [20,39], which measured this outcome, concluded that 60% of patients improved and 22% of patients recovered after treatment with CBT and GET. If that would be the case, then there should be a substantial reduction in the chronic fatigue syndrome symptom count. Figures for the end of treatment were not released, but at 52 weeks there was no statistically significant difference in the improvement in chronic fatigue syndrome symptom count between GET and SMC (*p* = 0.0916), GET and APT (*p* = 0.23) or between CBT and APT (*p* = 0.0986).

### 2.5. Reliance on One Subjective Symptom for Diagnostic Purposes

According to Flottorp et al. [5], “the NICE guideline committee presented a new non-validated diagnostic definition of CFS/ME, making post-exertional malaise (PEM) a required criterion. This reliance on one subjective symptom for diagnostic purposes is inconsistent with the guideline committee’s downgrading of trials that use subjective symptoms as primary endpoints”. Why Flottorp et al. talk about “*reliance on one subjective symptom*” is unclear because the NICE guideline committee itself wrote the following about this [1]. “The committee… recommended that ME/CFS should be suspected in people with these 4 key features:

1. Debilitating fatiguability that is not the result of ongoing excessive physical, emotional or mental exertion, and is not substantially alleviated by rest.
2. Post-exertional malaise, which is disproportionate to the amount of exertion (cognitive, physical, emotional and, social), and can be delayed.
3. Unrefreshing sleep. (The committee explained this in the following manner: “sleep difficulties are one of the central features of ME/CFS or a reversed sleep-wake cycle”).
4. Cognitive difficulties.

In addition to the four symptoms the committee agreed that as in the IOM 2015 criteria there should be a substantial reduction or impairment in pre-illness levels of function”.

Flottorp et al. [5] also refer to comments by the Royal College of Psychiatrists with regard to this (pp1250–1251). This College states that “Why is PEM give such prominence?” and “the premise of the Committee, that there is no ME/CFS without PEM/PESE as it is currently defined or measured, is incorrect” [74]. The committee responded to that in the following manner [74]: “PEM is widely acknowledged in specialist ME/CFS practice as being a characteristic feature of ME/CFS. The difficulty for interpreting the evidence is that in the trials that do not use a criteria that has PEM as essential (and therefore a 100% ME/CFS population)”. They also stated that “after considering the stakeholder comments the committee agreed to revisit the evidence for the intervention reviews further scrutinizing the information on PEM reported in the trials and the application of indirectness and relevance in the evidence. The excluded studies list was also re-examined to ensure any relevant information relating to PEM in the included studies were not missed. Unpublished data was not accepted for this analysis”.

Flottorp et al. also wondered why the main characteristic of ME/CFS is post-exertional malaise and not chronic fatigue. The name Myalgic Encephalomyelitis goes back to an outbreak of an unknown disease in the Royal Free Hospital in London in 1955. This outbreak was witnessed and documented by Dr Melvin Ramsay, the infectious disease specialist of that hospital [75]. He noted that the main characteristic of the disease was an abnormally delayed muscle recovery after trivial exertion, which over time has evolved into post-exertional malaise. Professor Knoop, one of the authors of Flottorp et al., wrote the following about that in 2008: “ninety-one of our 96 patients complained of post-exertional malaise, which some suggest is the main characteristic feature of CFS” [16].

Also, many CBT and GET studies, including Prins et al. [13] and White et al. [20], used the Oxford criteria. These criteria, which were created by a group of British doctors which included a number of psychiatrists [76] who are now leading proponents of CBT and GET for ME/CFS, rely on one subjective symptom only, i.e., chronic disabling fatigue, for diagnostic purposes. It is unclear why Flottorp et al. do not complain about the reliance on one subjective symptom by the Oxford criteria and the trials that use them, for example the PACE trial which they use as evidence. A major problem of the Oxford criteria is artificially inflating the efficacy of CBT and GET. That is achieved because, according to the American Agency for Healthcare Research and Quality (AHRQ) in 2016, “using the Oxford case definition results in a high risk of including patients who may have an alternate fatiguing illness or whose illness resolves spontaneously with time”. The AHRQ concluded that these criteria should therefore be retired [77]. A report commissioned by the American National Institute of Health came to a similar conclusion in 2014 [78,79]. Moreover, a large study by Baraniuk (*n* = 6175) [80] concluded that “the Oxford criteria were untenable because they inappropriately selected healthy subjects with mild fatigue and CIF [chronic idiopathic fatigue] and mislabeled them as CFS”.

Finally, post-exertional malaise is one of the best differentiators between ME⁄CFS and major depressive disorder. As noted by Belgian researchers in 2010, “the severe exacerbation of symptoms following exercise, as seen in ME⁄CFS patients, is not present in other disorders where fatigue is a predominant symptom such as depression, rheumatoid arthritis, systemic lupus erythematosus and multiple sclerosis” [81].

### 2.6. Safety

According to Flottorp et al. [5], “NICE did not recommend GET, claiming this treatment is ineffective and harmful, based on anecdotal evidence from patient group surveys and qualitative studies, which it preferred to the systematically assessed trial safety data. NICE ignored a summary provided to them in the consultation period of a now published meta-analysis of safety outcomes in the ten published trials of GET, suggesting that GET is safe so long as it is properly prescribed”.

The meta-analysis the authors refer to is a meta-analysis by White and Etherington, which assessed the safety outcomes in 10 GET trials [82]. Eight of those 10 trials were included in the amended Cochrane exercise review by Larun et al. [18] and one of the authors of Flottorp et al., (Brurberg), was also one of the four authors of that Cochrane review. In the amended review they concluded that “the impact of exercise therapy on serious adverse reactions is uncertain” because most studies did not report about safety or “because the certainty of the evidence is very low”.

In their Lancet article, Flottorp et al. [5], argue that people who have negative opinions about CBT and GET should not be part of the NICE guideline committee. In view of this, it is unclear why they do not mention that one of the two authors of the meta-analysis of safety outcomes (Professor White), was the principal investigator of 3 of the 10 studies in the meta-analysis. According to an article by Pautasso [83] entitled *Ten Simple Rules for Writing a Literature Review*, “this could create a conflict of interest: how can reviewers report objectively on their own work?” Moreover, White and Etherington [82] assessed safety in the following manner: “The measures of safety we report were: the number of participants withdrawing from treatment and the numbers dropping out of trial follow-up. Some trials also reported clinical global impression (CGI) change scores of overall health, but rarely interpreted the data regarding deterioration on this measure. Therefore, the third measure of safety we collated was the CGI [clinical global impression change scores of overall health]”. However, not everybody who deteriorated was included in that definition, as “the CGI score of 5 (“a little worse”) was not included in our measure of deterioration”. The authors also noted the following. “Sometimes trials had more than one possible control intervention, so a choice had to be made. For the FINE trial, the control intervention chosen was supportive listening, since by definition no one dropped out of GP care”. Yet according to the FINE trial itself [84], its objective was “to evaluate the effectiveness of home delivered pragmatic rehabilitation—a programme of gradually increasing activity designed collaboratively by the patient and the therapist—and supportive listening—an approach based on non-directive counselling—for patients in primary care with chronic fatigue syndrome/myalgic encephalomyelitis or encephalitis”. The FINE trial also stated that “our control condition is treatment as usual by the general practitioner” [85]. Consequently, the meta-analysis by White and Etherington defined safety in such a manner that not everybody who deteriorated after GET was counted as deteriorated.

The two studies that were not included in the Cochrane exercise review are the GETSET study by Clark et al. [86], which included the corresponding author of the meta-analysis, and a study by Windthorst et al. [87]. In the GETSET study, only 68% in the treatment group (guided graded exercise self-help plus specialist medical care or GES) and 74% in the no treatment control group (special medical care or SMC) had ME/CFS according to the Fukuda criteria. In reality it was most likely even lower than that, because post-exertional malaise, the main characteristic of ME/CFS, is only an optional requirement according to these criteria [88].

At baseline, patients had been ill for 46 (23–114, GES) and 42 months (25–99, SMC) and their level of physical activity was 120 (30–360, GES) and 185 min per week (75–570, SMC). Consequently, many patients were still in the aforementioned three years where the chances of recovering spontaneously are the highest. Also, judging by their level of physical activity, they were only very mildly affected at best. According to the study itself, “five patients would need to be treated for one to benefit from GES”. Yet the participant-rated positive change in CGI of their illness from baseline was 14% (GES) and 6% (SMC). Consequently, only 8% benefited subjectively from GES. Therefore, the authors should have concluded that 12 patients would need to be treated for one to subjectively benefit from GES. Moreover, “the physiotherapists reported that 43 participants (42%) adhered to GES completely or very well, 31 (30%) moderately well, and 30 (29%) slightly or not at all”. As noted by Kindlon, “a treatment cannot be considered safe if patients do not actually adhere to it” [89]. The figures presented by the physiotherapists suggest that in up to 59% of cases, the treatment was not very well tolerated by even mildly affected patients. Such a treatment cannot be described as safe and effective. It’s also unclear if the treatment actually led to objective improvement, as the study did not use an objective outcome measure. The GETSET authors [86] themselves noted the following about that. “All outcomes were self-rated, which might lead to bias by expectation,…We did not measure any objective outcomes, such as actigraphy, which might have tested the validity of our self-rated measures of physical activity”. Consequently, the GETSET authors themselves are not sure if graded exercise therapy actually led to real improvement or not.

The study by Windthorst et al., (*n* = 28) compared GET to heart rate variability biofeedback therapy [87]. The latter is “a treatment method that includes cognitive and behavioural strategies”. Patients were self-referred, but male patients were excluded and “57 individuals cancelled their participation because of the treatment frequency and training intensity”. Consequently, patients who were not able to do the graded exercise program withdrew from taking part from the study before it had started. Therefore, only people who were only mildly affected and thought that the treatment would not be a problem for them took part, yet despite that, 27% dropped out from the GET group versus 0% from the control group. As Windthorst et al. concluded, “if the combination of both treatments would be superior to each of its components or to placebo remains unclear”. Consequently, neither Windthorst et al. nor the GETSET study present any evidence for the safety of GET, nor for its efficacy.

Finally, Flottorp et al. [5], do not mention the study by the Oxford Brookes University [90] (*n* = 2274) from 2019 on the safety of CBT and GET which was commissioned by NICE as part of their review of the ME/CFS guidelines. Worsening of symptoms after treatment was reported by 58.3% (CBT, which incorporates an element of GET in ME/CFS) and by 81.1% (GET). In addition, the percentage of patients who were bedridden and dependent on help from others due to severe ME/CFS increased from 12.6% to 26.6% after treatment (CBT, which incorporates an element of exercise therapy in ME/CFS) and from 12.9% to 35.3% (GET). The very high dropout rate of 55%, 73%, and 80% at 6, 9, and 12 months, respectively, in the evaluation study of a 12-month program of GET in a sports medical department of a Dutch hospital [91], which we discuss in more detail in Appendix A, confirms the findings of the Oxford Brookes University and the unsuitability and harmfulness of GET as a treatment for ME/CFS.

## 3. Discussion

In a commissioned article in *The Lancet*, Flottorp et al. [5] criticize the NICE guideline committee for dropping CBT and GET which, according to them, are the only two evidence-based treatments available for ME/CFS. They accuse the committee of “selective use of the evidence from randomised studies, cherry-picking statements from qualitative studies, and relying on the opinions of the committee”. They also state that “NICE disregarded the best available research evidence and tarnished the guideline process”. And they finish by concluding that “this guideline denies patients treatments that could help them, undermines NICE as an international authority in guideline development, and jeopardizes fundamental scientific principles by allowing some processes driven by ideology”. Furthermore, according to Flottorp et al., since the previous NICE guideline (2007), more trials have provided evidence for the efficacy of CBT and GET. Flottorp et al. use three trials, which includes the PACE trial and the Dutch FITNET study, two Cochrane reviews, and a meta-analysis to support this. However, there were serious problems with these studies and reviews. The non-blinded PACE trial, for example, not only relied on subjective primary outcomes, but it also made an extensive number of endpoint changes that created an overlap in entry and recovery criteria. Consequently, 13.3% of patients who were classed as ill enough to take part were also already classed as recovered according to one or two of the primary outcomes, that is, before they had received any treatment. Something like that should not happen in a properly conducted study and those patients should have been excluded from the PACE trial.

Problems with the Dutch FITNET study included not publishing its objective outcome (actometer). The authors acknowledged later that Internet-based CBT does not lead to objective improvement. Moreover, they also used a post-hoc definition of recovery. The definition of recovery from both the PACE trial and the FITNET study was so broad that it included the severely ill, and both trials ignored the null effect of their objective outcomes. Flottorp et al. also use two Cochrane reviews as evidence but do not mention that The Cochrane Library itself has put up notices on their website that both the CBT and GET reviews are outdated, should not be used, and need a major overhaul.

Flottorp et al. also argue that the committee should have relied on the subjective outcomes from the CBT and GET studies. This is puzzling for a number of reasons. First of all, the basis of the CBmodel as an explanation for ME/CFS is that patients interpret their symptoms incorrectly. It is illogical and unscientific to then rely on patients interpreting their symptoms by using subjective outcomes because that would imply that patients are interpreting their symptoms wrongly but at the same time they are also interpreting the same symptoms rightly. Moreover, CBT and GET studies are non-blinded by definition, and relying on subjective outcomes in those studies is very prone to all sorts of different forms of bias. Consequently, one could get the impression of efficacy, even though patients have not benefited from the treatment. An additional problem of the CBT studies is that patients are instructed to interpret their symptoms differently. A subjective improvement could then simply be caused by answering questionnaires in a different way. This is also known as response shift bias, and the only way to correct for this is by using objective outcomes.

Moreover, the GETSET study [86], one of the studies in the meta-analysis by White and Etherington [82], which was used by Flottorp et al. [5] as evidence, noted the following about using subjective outcomes: “All outcomes were self-rated, which might lead to bias by expectation”. But also that “objective outcomes, such as actigraphy,…might have tested the validity of our self-rated measures of physical activity”. Finally, if Flottorp et al. want to rely on patient reported outcomes, then why do they ignore that patients have been saying for a long time that CBT and GET are ineffective and also harmful?

Flottorp et al. [5] complain that some committee members and two of the three expert witnesses have expressed a negative opinion about CBT and GET on social media, yet our analysis shows that they do not provide any evidence for that (see Appendix A). They also do not mention that it was a committee consisting of 21 members nor do they mention that patients were worried because many of the members of that committee were known to favour CBT and GET. It also raises the question as to why Flottorp et al. do not complain about the members of the committee who had expressed a positive opinion about these treatments. They also do not complain about the fact that all five authors of their own article have expressed strong opinions about the efficacy of CBT and GET for ME/CFS over the years. Moreover, at least two of them have also successfully built a career based on that. It is also unclear why they do not complain about the potential conflict of interests of a meta-analysis by White and Etherington [82], which they use as evidence that graded exercise therapy is safe, because one of the two authors of that meta-analysis is not only one of the leading proponents of CBT and GET for ME/CFS. But he is also the principal investigator of three of the 10 studies in that meta-analysis. According to this meta-analysis, exercise is safe, yet the Cochrane exercise reviewers, who reviewed eight of those 10 studies, concluded that most studies did not report on the safety of GET, and therefore one doesn’t know if GET is safe or not. Moreover, our analysis shows that the remaining two of those 10 studies do not provide any evidence that GET is safe. Nor do they provide any evidence that GET is effective. For example, the GETSET study by Clark et al., did not use an objective outcome measure, so they could not test “the validity of our self-rated measures of physical activity”, according to the authors themselves. Moreover, 12 mildly affected patients needed to be treated in the GETSET study for one to have a subjective improvement of their ME/CFS, and up to 59% of participants in this study did not adhere to the treatment according to the trial itself. This might suggest that GET is not safe for mildly affected patients either.

Furthermore, the authors, including the authors of the aforementioned meta-analysis by White and Etherington, dismiss patient surveys which for decades have shown that both treatments are not safe, because they are based on subjective observations from patients. Yet at the same time they want to rely on subjective observations from patients, i.e., subjective outcomes, in CBT and GET trials.

Furthermore, examining important subjective outcomes for patients from these CBT and GET studies, for example CFS symptom count and quality-of-life scores, shows that CBT and GET do not lead to substantial subjective improvement.

An influential systematic review by Whiting et al. from 2001 [56], concluded that one of the reasons why subjective outcomes in those studies are unreliable is because patients are better able to cope but haven’t made any actual improvement. Whiting et al. advocated for the use of objective outcomes like objective physical performance and activity and/or work status. An analysis of CBT and GET trials that use those outcomes shows that both treatments do not lead to improvement of objective physical activity/fitness as measured via the actometer, six-minute walk test, step test, cardiopulmonary exercise testing, etc. Moreover, an extensive review found that both treatments have a negative effect on work status rather than a positive one.

Professor Ioannidis [92] noted a number of important things in a famous article about research findings, including the following.

1. “The greater the financial and other interests and prejudices in a scientific field, the less likely the research findings are to be true”.
2. “The greater the flexibility in designs, definitions, outcomes, and analytical modes in a scientific field, the less likely the research findings are to be true”.

Examples of this in the evidence presented by Flottorp et al., are the aforementioned extensive number of endpoint changes in the PACE trial, which created an overlap in entry and recovery criteria and the post-hoc definition of recovery in the FITNET trial, which included the severely ill. Professor Ioannidis also concluded that “investigators working in any field are likely to resist accepting that the whole field in which they have spent their careers is a “null field”. This might explain the reluctance by Flottorp et al. and other proponents of the CBmodel to accept the conclusions by the NICE guideline committee.

One of the main proponents of CBT for ME/CFS is Dutch internist Professor Van der Meer. He was part of the expert Working Group which in 2017 helped the European Academies Science Advisory Council (EASAC) to prepare a statement on homeopathy [93]. This study is of particular interest for the current discussion because they made a number of recommendations, including “there should be consistent regulatory requirements to demonstrate efficacy, safety and quality of all products for human and veterinary medicine, to be *based on verifiable and objective evidence*, commensurate with the nature of the claims being made. *In the absence of this evidence*, *a product should be neither approvable nor registrable* [italic by us] by national regulatory agencies for the designation medicinal product”. And the same should apply to treatments like CBT and GET.

They also recommended that “evidence-based public health systems should not reimburse homeopathic products and practices unless they are demonstrated to be efficacious and safe by rigorous testing”. If that had been applied to CBT and GET, then these treatments would never have been recommended by guidelines all over the world. Why professor Van der Meer, for example, applied these standards to homeopathy studies but not to (his own) CBT studies, is unclear.

Flottorp et al. accuse the committee of:

- selective use of the evidence from randomised studies,
- cherry picking statements from qualitative studies,
- relying on the opinions of the committee,
- disregarding the best available research evidence and tarnishing the guideline process,
- undermining NICE as an international authority in guideline development,
- jeopardizing fundamental scientific principles by allowing some processes driven by ideology. Yet, our analysis of the arguments and evidence presented by Flottorp et al., didn’t find any evidence that the NICE guideline committee did that.

Finally, Flottorp et al. [5] emphasise the need to listen to patients, yet they continue to ignore that patients have been saying for a long time that CBT and GET are neither safe nor effective. As noted by psychologist professor Kunst, the following “can’t be stressed enough: The reason why we lack treatments for #LongCovid is that patients of similar post-viral conditions have been gaslit and disbelieved for decades (i.e., #MECFS). The long-term effects of viruses are nothing new—it is just that nobody seemed to care” [94].

## 4. Conclusions

Flottorp et al. state that they present evidence in their article in *The Lancet* that the NICE guideline committee came to the wrong conclusion. However, our analysis shows that the studies used by the authors in an effort to support their claim of efficacy of CBT and GET in reality not only show the opposite but also highlight the serious problems, for example, with the design and the reporting of the results of these studies. These problems included relying on subjective primary outcomes in non-blinded studies, extensive endpoint changes, selective reporting, ignoring and/or not publishing the null effect of their own objective outcomes, selecting patients who didn’t have the disease under investigation or had a self-limiting disease and labelling the severely ill as recovered. Our analysis also confirms that NICE was right in dropping CBT and GET as treatments for ME/CFS.

## Appendix A. Other Important Evidence Presented by Flottorp et al.

### Appendix A.1. Joint Statement by Seven Medical Colleges

According to Flottorp et al. [5], “seven medical leaders in Royal Colleges and faculties in the UK declared in a joint statement: “there is considerable disquiet in the medical profession and some patient groups about the way the data and evidence have been assessed”.

The wording, “*some patient groups*” suggests that more than one patient group is not happy about that. It is unclear why the authors do not mention which patient groups they mean, as we are not aware of any patient group, let alone more than one patient group, who are unhappy about that.

Seven Colleges signed the joint statement in response to the NICE guidance on ME/CFS. The Colleges want to continue to use CBT and GET but their statement doesn’t provide any evidence for the efficacy of these two treatments [95]. The Colleges claim that “Graded Exercise Therapy as defined in the guidance is not reflective of the personalised paced exercise programmes that are currently used in the NHS and termed GET”. If that was the case, then there would be no reason for the seven colleges to complain about dropping GET because they are not using it in the first place. Moreover, if they are using *Graded Exercise Therapy redefined as personalised paced exercise programmes*, then that would also mean that they are using a treatment that hasn’t been trialed and tested. Consequently, that would mean that the seven colleges are using non-evidence-based medicine. However, we think it is reasonable to assume that they don’t mean that because the seven colleges would not be using non-evidence-based medicine.

Why the seven colleges, which included the Faculty of Sport and Exercise Medicine, ignore the evaluation report by Dutch sports physicians of the efficacy of GET for ME/CFS in their own sports medical department of a Dutch hospital, is unclear. Van Berkel et al. [91] reported dropout rates over the course of 12 months of treatment with GET in that hospital of 33%, 59%, and 72% after 6, 9, and 12 months, respectively. However, 64 patients who had dropped out of treatment before the three-month assessment, and who had not just started treatment, had not been referred to another specialty, or had not recovered, were excluded from the analysis. Yet, if one wishes to determine the effectiveness of a treatment and how well it is tolerated, then one should not exclude such patients. If they had been included, then the dropout rate after 6, 9, and 12 months would have been 55%, 73%, and 80%, respectively. At baseline, 66.7% of patients in this study were working or studying, on average, 28.4 h per week. This suggests that many patients in the study were only very mildly affected. Consequently, it seems reasonable to assume that GET at 50 to 60% of a patient’s exercise capacity was not very well tolerated or safe for these patients either. The inappropriateness of GET, even for very mild ME/CFS, is further highlighted by the fact that patients were treated with a personalised GET programme which the seven colleges state they use.

Finally, the three principal investigators of the PACE trial [96] stated the following in a letter published in a British newspaper two months after Flottorp et al. published their article in *The Lancet*. “Nice has recently advised that GET should not be offered and CBT only used to reduce distress, but four of the medical Royal Colleges did not endorse this advice as they considered Nice had made errors when reviewing the evidence”. The authors do not mention the names of those colleges nor do they provide a reference for that. Their statement however suggests that three of the seven colleges mentioned by Flottorp et al. have now changed their mind and no longer disagree with NICE.

### Appendix A.2. Conflicts of Interests and Negative Opinions

According to Flottorp et al. [5], “we know from social media that some of the committee members and two of the three expert witnesses had negative opinions regarding the interventions”. Their two references are the list of three expert witnesses published by NICE and a thread on the Science for ME forum which was 43 pages long at the time of writing [97]. It is unclear why Flottorp et al. do not use quotes so that people can understand what they complain about. It is also unclear why they refer to a thread on a forum and not to the posts on that thread they complain about. If one reads the posts on that thread on the Science for ME forum, then patients on that thread are actually complaining about the opposite, as the following three comments from that thread show.

1. “With a couple of exceptions, the professional members look pretty bad to me” [98]
2. “Having so many BPS committee members” [99]
3. “How on earth can such a heavily BPS weighted GDC produce useful guidelines?” [100]

Moreover, if *some of the committee members* had negative opinions, then that raises the question of how many members sat on the committee. According to the Committee membership list, the Guideline Committee had 20 members and one co-opted member [101]. It also raises some other questions. For example, are Flottorp et al. [5] not concerned about the fact that:

1. some committee members had expressed positive opinions?
2. five of the five authors of their own article had expressed positive opinions?
3. at least two of the five authors of their own article have successfully built a career, became professors and are seen as ME/CFS experts, because of expressing those positive opinions?

Flottorp et al. [5] also complain about two of the three expert witnesses without making it clear which two they refer to and what those negative opinions regarding the interventions actually are. According to Appendix 3 of the NICE guidelines [102], the expert testimonies are from the following three doctors. One expert witness is a consultant liaison psychiatrist (Dr Mujtaba Husain) who offers CBT and GET in the persistent physical symptoms research and treatment unit formerly known as the chronic fatigue research and treatment unit. The multidisciplinary team of that unit only consists of “cognitive behavioural therapists, clinical psychologists, physiotherapy and consultant psychiatrists”. ME on the other hand has been classified as a neurological disease by the World Health Organisation (WHO) since 1969 [103], with CFS as an equivalent, which is not reflected in the name of the unit or the composition of the multidisciplinary team. Why do the authors not complain about that? Might it be because this expert witness has a positive view of the treatments favoured by Flottorp et al.? Does it also mean to say that a positive view by expert witnesses is okay according to Flottorp et al., but a negative one is not?

The second expert witness (Dr Nina Muirhead) is a dermatological surgeon who became ill with ME/CFS in 2016 and as a doctor did not recognise the illness herself. Moreover, 12 other doctors did not recognise it either. Dr Muirhead did not post anything on that forum thread, so it is unclear what negative comments by Dr Muirhead, Flottorp et al. refer to and as far as we could tell, she is not a member of that forum either.

The third expert witness is Professor Jonathan Edwards, a retired rheumatology professor who pioneered the use of Rituximab for rheumatoid arthritis. A forum member wrote on the aforementioned thread, “it is also misleading to refer to CBT & GET as ‘treatments’ of choice’. They cannot properly be described as treatments, since, as NICE admits, they do not address the core pathology of ME” [104]. Professor Edwards [105] responded to that by saying, “the argument here is not valid. Treatments very often do not address core pathology but work very well—like anti-TNF for rheumatoid arthritis”. And when forum members were complaining about the fact that the NICE guideline committee was biased, Professor Edwards responded by saying, “I think this is a good opportunity for S4ME [the Science for ME forum] to demonstrate its scientific and ethical credentials by making a well-reasoned statement” [106]. In another comment, he noted that “the one thing to keep away from is this business of psychological versus physical” [107]. These comments seem to be very neutral and the opposite of negative. It’s therefore unclear why Flottorp et al. complain about his comments. Professor Edwards has subsequently responded to the accusations in *The Lancet* article by Flottorp et al. The following are a few extracts from his responses [108,109].

“I was unimpressed by the transparent insinuation that as an expert witness to the NICE committee that my view was biased—presumably reflecting some imagined vested interest. None of this matters to me—I am a retired rheumatologist who studies Leibniz—but it matters to standards in medical science. The vested interests are on your side—including direct interest in the therapies and research and in the reputation of Cochrane reviews such as those co-authored with Larun”.

“The reasons why the NICE committee were right to downgrade evidence on therapist-delivered treatments are clearly set out in my expert witness testimony. There is nothing difficult about the arguments. They reflect established fact and common sense. In my presentation, I included a slide, carrying Cochrane approval, indicating the main point—that ‘If you are reading a study that is un-blinded, with subjective outcome measures, then you may as well stop reading it and move on.’ My only regret is that at the time I had not seen Heins et al., (2013) J. Psychosomatic Res. 75, 3, 235–241 which crystallizes how naive investigators are in this field. The suggestion seems to be that people feel better because they believe they are doing more—when in fact they aren’t. Any sensible person can see how ridiculous this is as a useful outcome. And a lot of people with ME/CFS are sensible”.

“The change from 2007–2021 is simple—the evidence now indicates that there is no worthwhile effect—as emphasised at the NICE Round Table discussion”.

“What has shocked me most is how pervasive the lack of rigor is in the Cochrane circuit for non-pharmacological treatment”.

“I also think an apology might be due for implying that somehow as an expert for the NICE committee I was prejudiced in my opinion. Readers of this thread will see that my approach has been purely to look at evidence quality with no bias towards one thing or another.

The irony is that the charge of competing interest rests entirely on those writing *The Lancet* comment. I have no competing interest at all.

My opinion of the PACE trial was not influenced by patients. It was created by listening to Dr Peter White [the main principal investigator of the PACE trial] presenting his view of patients being anti-science to an audience including a lot of patients. (Tact was not a strong point.) Starting off by flashing up a single data slide with a truncated Y axis and a minuscule difference in subjective scores between test and control told me all I needed to know”.

### Appendix A.3. Cross-Over during Long-Term Follow-Up

According to Flottorp et al. [5], “the guideline committee decided to consider trial outcomes at the furthest time away from recruitment, overlooking data on end of treatment and the trial primary endpoint in the largest trial of CBT and GET. This omission contributed to downgrading for imprecision and the questionable conclusion in the new guideline that there was insufficient evidence for the efficacy of CBT and GET. Here, NICE did not account for cross-over between the intervention and the control group during long-term follow-up”.

First of all, according to the PACE trial, the largest trial of CBT and GET, which was used by Flottorp et al. as evidence, itself, “primary outcomes were fatigue…and physical function…up to 52 weeks after randomization” [20]. Also, fatigue and physical functioning scores were better after 52 weeks than at the end of treatment at 24 weeks, as can be seen in Table A1. However, Table A1 also shows that the mean fatigue and physical functioning scores were still so low that patients were still ill enough to enter the same trial again and get the same treatment again after treatments deemed to be effective by the trial itself.

**Table A1:** fatigue and physical functioning scores after CBT and GET in the PACE trial.

	Fatigue		Physical Functioning	
Time	CBT	GET	CBT	GET
24 wks	21.5	21.7	54.2	55.4
52 wks	20.3	20.6	58.2	57.7
Entry score requirement	18 or more	18 or more	65 or less	65 or less

24 wks: end of treatment; wks: weeks. Source: PACE trial [20].

As far as NICE not accounting for crossover between the intervention and the control group during long-term follow-up, a review of the PACE trial noted the following about crossover at long-term follow-up in that study [53]. First of all, “researchers cannot control the environment subsequent to completion of a trial; therefore, an effect cannot be attributed to the receipt of any form of additional post trial treatment. Another problem is the carryover effect, whereby the effect of a treatment is carried over to the second phase, which in this case was the phase following trial completion. As noted by Larun et al. [the Cochrane exercise reviewers] this is a greater problem “when the condition of interest is unstable,” and “both effects are very likely in CFS/ME”. The same review of the PACE trial also noted that “the Supplementary Appendix of the PACE trial’s long-term follow-up article, which “formed part of the original submission”, was “peer reviewed” and “supplied by the authors”, shows that the majority of participants, i.e. 76% and 83%, did not have any additional CBT respectively GET, after the trial had finished. Last but not least, the Supplementary Appendix long-term follow-up shows that in all 4 groups, patients who did not receive additional treatment subsequent to trial completion exhibited lower fatigue and higher physical functioning scores relative to those of patients who received additional treatment. This suggests that additional CBT and GET, provided by qualified and experienced therapists from the trial subsequent to trial completion, could have been detrimental to patients’ health. And on top of that, in the CBT and SMC groups an adequate number of sessions of CBT and GET were more detrimental than an inadequate number of sessions”.
